# Supplementary material for: Cancer patient survival can be parametrized to improve trial precision and reveal time-dependent therapeutic effects
Source: Nat Commun. 2022 Feb 15;13:873. doi: 10.1038/s41467-022-28410-9 (PMC8847344; doi:10.1038/s41467-022-28410-9)
Supplement: Supplementary file 5 — Supplementary Dataset 2 [file 41467_2022_28410_MOESM5_ESM.zip › Supplementary Data File 2/Delta alpha vs beta plots- Fig 6, 7, S9/Delta alpha beta SEFS.pdf]

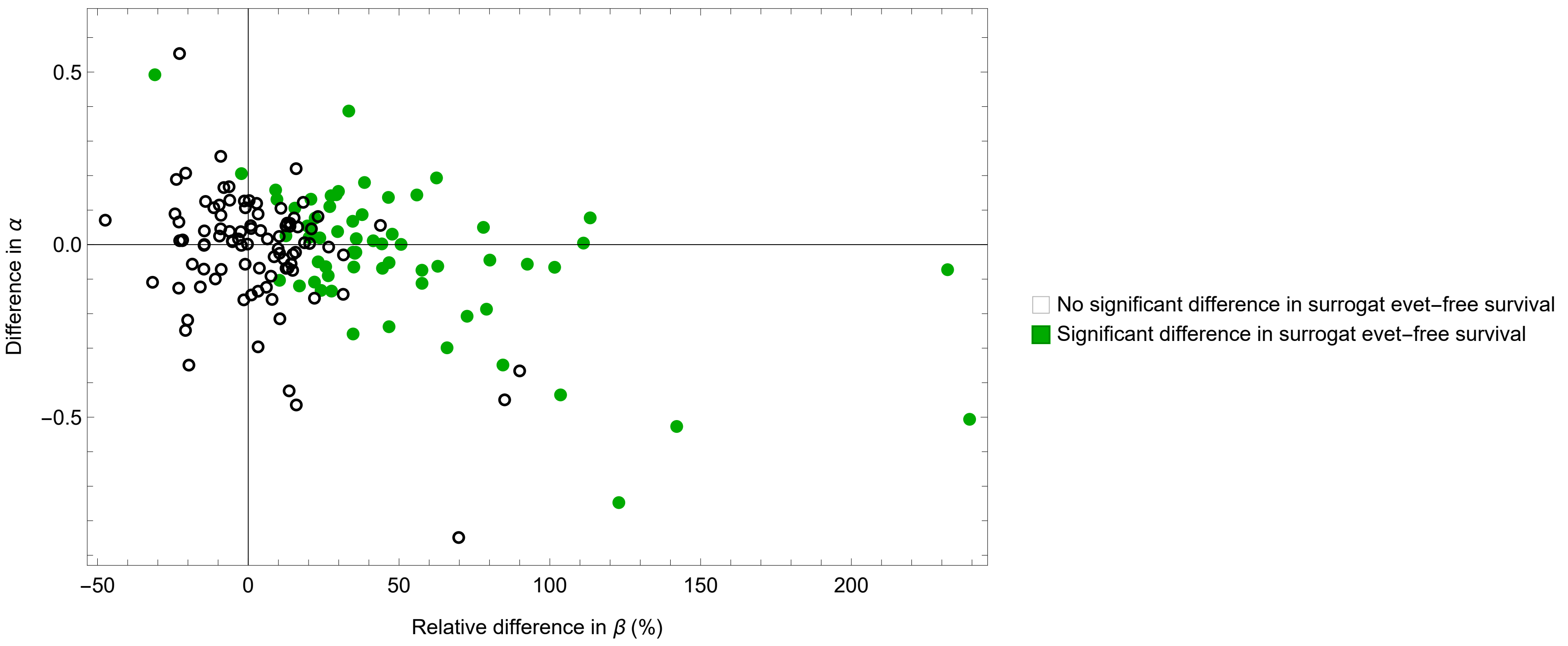
0.5
0.0
−0.5
Difference in α
−50
0
50
100
150
200
Relative difference in β (%)
No significant difference in surrogat evet−free survival
Significant difference in surrogat evet−free survival
